# Serum neurofilament light chain and initial severity of neurological disease predict the early neurological deterioration in Wilson’s disease

**DOI:** 10.1007/s13760-022-02091-z

**Published:** 2022-09-13

**Authors:** Tjalf Ziemssen, Lukasz Smolinski, Anna Członkowska, Katja Akgun, Agnieszka Antos, Jan Bembenek, Iwona Kurkowska-Jastrzębska, Adam Przybyłkowski, Marta Skowrońska, Barbara Redzia-Ogrodnik, Tomasz Litwin

**Affiliations:** 1 Center of Clinical Neuroscience, Department of Neurology, University Clinic Carl Gustav Carus and Dresden University of Technology, Dresden, Germany; 2 Second Department of Neurology, Institute of Psychiatry and Neurology, Sobieskiego 9, 02-957 Warsaw, Poland; 3 Department of Gastroenterology and Internal Medicine, Medical University of Warsaw, Warsaw, Poland; 4 Department of Radiology, Institute Psychiatry and Neurology, Warsaw, Poland

**Keywords:** Wilson’s disease, Serum neuro-filament light chain, Magnetic resonance imaging, UWDRS, Neurological deterioration

## Abstract

**Background:**

In Wilson’s disease (WD), early neurological deterioration after treatment initiation is associated with poor outcomes; however, data on this phenomenon are limited. Our study analysed the frequency and risk factors of early neurological deterioration in WD.

**Methods:**

Early neurological deterioration, within 6 months from diagnosis, was defined based on the Unified Wilson’s Disease Rating Scale (UWDRS): any increase in part II or an increase of ≥ 4 in part III. In total, 61 newly diagnosed WD patients were included. UWDRS scores, brain magnetic resonance imaging (MRI) scores, copper metabolism parameters, treatment type and serum neuro-filament light chain (sNfL) concentrations at diagnosis were analysed as potential risk factors of early deterioration.

**Results:**

Early neurological deterioration was observed in 16.3% of all WD patients; all cases of worsening occurred in the neurological phenotype (27.7%). Higher scores were seen in those who deteriorated compared with those who did not for UWDRS part II (4.3 ± 5.0 vs 2.0 ± 5.9; *p* < 0.05), UWDRS part III (21.5 ± 14.1 vs 9.3 ± 16.4; *p* < 0.01) and MRI-assessed chronic damage (3.2 ± 1.6 vs 1.4 ± 2.2; *p* = 0.006); all these variables indicated the initial severity of neurological disease. Pre-treatment sNfL concentrations were significantly higher in patients who deteriorated compared with those who did not (33.2 ± 23.5 vs 27.6 ± 62.7 pg/mL; *p* < 0.01). In univariate logistic regression amongst all patients, chronic damage MRI scores, UWDRS part III scores and sNfL concentrations predicated early deterioration. In the neurological WD, only sNFL were a significant predictor. In bivariate logistic regression amongst all patients, sNfL remained the only significant predictor of deterioration when corrected for MRI scores.

**Conclusion:**

sNfL concentrations are a promising biomarker of the risk of early neurological deterioration in WD.

## Introduction

Wilson’s disease (WD) is an inherited disorder of copper metabolism in which copper tissue accumulation, mainly in the liver and brain, results in a range of clinical symptoms [1–3]. By correcting the copper body balance, WD can be treated successfully with pharmacological agents, such as chelators or zinc salts. With early diagnosis and appropriate treatment, WD has a favourable outcome in almost 85% of patients [4–11]. However, in some WD patients, despite the introduction of anti-copper treatment, so-called paradoxical neurological deterioration can occur at treatment initiation, often leading to an adverse treatment outcome or even death due to complications of immobilisation [12–14]. Data on the frequency of paradoxical early neurological deterioration are conflicting. Initially, early neurological deterioration was reported in as many as 50% of WD patients on chelators [1], but more recent publications report a lower frequency of 11.6% [14]. In the search for new effective treatments for WD, decreasing the risk of early neurological deterioration remains one of the most important goals [15, 16].

Only a limited number of published papers have used objective neurological scales to analyse early neurological deterioration [14, 16, 17], and there is a need to define neurological deterioration as well as establish risk factors to determine which patients should be more closely monitored. Previous studies which investigated the potential risk factors of early neurological deterioration in WD focussed on clinical signs and symptoms of WD, age of their onset, type of neurological symptoms, concomitant medications use, presence of chronic liver disease [16, 17]. In the last two years, there was a progress in neuroimaging as well as in studies evaluating brain injury biomarkers in WD [18–20]. The new semiquantitative tools for neurological WD monitoring have been proposed recently, namely a brain magnetic resonance imaging (MRI) semiquantitative scale [18] and measurement of the biomarker of brain injury, serum neuro-filament light chain (sNfL) [19, 20].

The aim of our study was to determine the frequency of early neurological deterioration in WD and to assess the ability of existing and new neurological tools to predict its occurrence.

## Methods

This was a prospective analysis of all consecutive newly diagnosed WD patients admitted to the Second Department of Neurology, Institute of Psychiatry and Neurology, Warsaw, Poland, between June 2012 and June 2017. The study is part of a project assessing the significance of sNfL as a biomarker in WD patients conducted by the Institute of Psychiatry and Neurology, Warsaw, Poland and the Center of Clinical Neuroscience, Department of Neurology, University Clinic Carl Gustav Carus & Dresden University of Technology, Dresden, Germany. The study was approved by the local Bioethical Committee of the Institute of Psychiatry and Neurology, Warsaw; all study participants gave informed written consent.

A diagnosis of WD was established based on clinical symptoms, copper metabolism and genetic examination using international criteria (Ferenci score) [2]. The disease was considered symptomatic if the patient presented with clinical symptoms at the time of diagnosis. Patients were classified according to phenotypic presentation as neurological (with neurological symptoms), hepatic (without neurological symptoms) or presymptomatic, based on international criteria [2].

All patients were examined neurologically using the Unified Wilson’s Disease Rating Scale (UWDRS) [20]. In addition, at the time of diagnosis, all patients underwent brain MRI using the Philips Achieva 1.5 T system (Philips Healthcare, Eindhoven, Netherlands), with sequences as described previously [18]. All MRI examinations were analysed by a blinded neurologist (BRO) using a semiquantitative scale for assessing brain MRI abnormalities in WD, which provides scores for acute toxicity, chronic damage and a total score [18].

Copper metabolism parameters (serum ceruloplasmin and copper concentrations as well as daily urinary copper excretion) and sNfL concentrations were analysed in the same laboratory. Serum ceruloplasmin concentrations were assessed using a colorimetric enzymatic assay, whilst serum and urine copper concentrations were analysed by atomic absorption spectroscopy as described previously [3]. sNfL concentrations were determined in plasma samples collected at diagnosis using the SimplePlex™ NfL Assay (ProteinSimple, CA, USA) and Ella™ instrument, according to the manufacturers’ instructions and as previously described, with a lower limit of quantification of 2.7 pg/mL [21].

Routine visit protocol for newly diagnosed WD patients in our centre consists of: the baseline—first visit at diagnosis. Next visits are performed after 3 months, 6 months, and every 6 months up to 2 years after diagnosis. Then we perform visits once a year, unless there are some healthy problems, then visits are more frequently performed.

Tests which we perform, at each visits are: copper metabolism (serum cerulopasmin and copper levels, urinary copper excretion), liver function tests, haematology, urine samples (especially in d-penicillamine treated patients) as well as coagulogram. Each patient is examined by well-trained and experienced neurologist and always UWDRS part II and III are completed, as well as neurological examination. Each year we perform ultrasound examination of abdomen, and at diagnosis 2 years later, we perform brain MRI examination.

Additionally we collect patients’ serum for future studies (which was approved by Bioethical Committee).

For purpose of this study, all patients after diagnosis and anti-copper treatment introducing [d-penicillamine or zinc salts (Table 1)] were followed up 6 months after diagnosis using the UWDRS to detect cases with neurological deterioration during this period. Neurological deterioration was defined as any deterioration in UWDRS part II or/and increase of at least 4 points in UWDRS part III as previously described [14]. Both groups of patients, those with and without deterioration, were analysed according to selected laboratory, neuroradiological and clinical variables.

**Table 1:** The impact of selected baseline clinical and laboratory parameters on early neurological deterioration

Characteristic	Without early neurological deterioration (*n* = 51)	With early neurological deterioration (*n* = 10)	*P* value
Gender, male, *n* (%)	29 (56.8%)	3 (30.0%)	0.170
Age at onset, mean ± SD (years)	28.1 ± 10.9	30.5 ± 11.6	0.575
Age at diagnosis, mean ± SD (years)	31.2 ± 11.1	36.2 ± 14.3	0.267
Neurological form of WD (*n* = 36)	26 (50.9%)	10 (100%)**	0.003
Hepatic form of WD (*n* = 18)	18 (35.2%)	0 (0%)**	0.026
Pre-symptomatic form of WD (*n* = 7)	7 (13.7%)	0 (0%)	0.587
Diagnostic delay*, mean ± SD (years)	2.4 ± 3.6	5.7 ± 9.2	0.088
d-penicillamine, *n* (%)	22 (43.2%)	6 (60%)	0.327
Zinc sulphate, *n* (%)	29 (56.8%)	4 (40%)	0.327
UWDRS part II score, mean (range)	2.0 (0–38)	4.3 (0–16)**	0.033
UWDRS part III score, mean (range)	9.3 (0–96)	21.5 (2–41)**	0.002
MRI acute toxicity score, mean (range)	1.5 (0–9)	3.3 (0–11)	0.071
MRI chronic damage score, mean (range)	1.4 (0–10)	3.2 (0–5)**	0.006
MRI total score, mean (range)	3.0 (0–12)	6.5 (0–13)**	0.020
Serum ceruloplasmin concentration, mean ± SD (mg/dL)	13.0 ± 6.6	11.9 ± 6.7	0.718
Urinary copper excretion, mean ± SD (μg/24 h)	373.5 ± 1220.0	195.9 ± 184.0	0.640
sNfL concentration, mean ± SD (pg/mL)	27.2 ± 62.7	33.2 ± 23.5**	0.006

^*^The time between first symptoms onset and disease diagnosis

^**^Denotes statistically significance

*MRI* magnetic resonance imaging, *SD* standard deviation, *sNfL* serum neuro-filament light chain, *UWDRS* Unified Wilson’s Disease Rating Scale

## Statistical analysis

Data are presented as percentages or as means with ranges and standard deviations (SD). Comparisons were made using the Fisher’s exact test or the Mann–Whitney *U* test as appropriate. Receiver operating characteristic (ROC) curve analyses were used to compare the diagnostic performance of selected variables in distinguishing patients with or without early neurological worsening. Areas under the ROC curves (AUCs) and optimal cut-offs were calculated based on the Youden’s statistic. Then, sensitivity, specificity, positive predictive value (PPV), and negative predictive value (NPV) were calculated for the respective cut-offs. Univariate logistic regression models were used to calculate odds ratios (ORs) of having an early neurological worsening for selected predictors. For the predictors analysed by ROC curves, the patients with values above the respective cut-offs were compared with those having values below the cut-offs in all patients as well as in patients with neurological presentation only. Additionally, the independence of individual predictors was analysed in bivariate logistic regression models. Statistical analyses were performed with Statistica 13.3 (Stat Soft Inc, 2020 Tulsa OK, USA), R v.3.6.3 or JASP v.0.16. The pROC package was used for ROC curve analyses [22]. *p* < 0.05 was considered statistically significant.

## Results

Out of 61 newly diagnosed WD patients, 10 patients (16.3%) had early neurological deterioration during first 6 months of treatment. All 10 patients presented with neurological symptoms at the time of diagnosis. The frequency of deterioration in the neurological form was 27.7% (10/36). The demographic, clinical as well as laboratory, and neuroradiological characteristics of patients presenting with or without deterioration are presented in Table 1. Patients who experienced early deterioration had more severe neurological impairment scored by UWDRS part II (4.3 ± 5.0 pts vs 2.0 ± 5.9; *p* < 0.05) and part III (21.5 ± 14.1 vs 0.9.3 ± 16.4; *p* < 0.01) (Table 1). Additionally patients who experienced neurological deterioration had initially more severe brain MRI chronic brain damage score (3.2 ± 1.6 vs 1.4 ± 2.2) and total brain score (6.5 ± 4.3 vs 3.0 ± 4.1 pts) (*p* < 0.01), but not acute toxicity score. The initial sNfL concentrations were significantly higher in patients who deteriorated (33.2 ± 23.5 pg/mL vs 27.6 ± 62.7 pg/mL; *p* = 0.01). Additionally, there was statistically significant trend for longer WD disease latency as a risk factor of neurological deterioration 5.7 ± 9.2 vs 2.4 ± 3.6 years (*p* = 0.088). The type of WD treatment (d-penicillamine or zinc salt), gender as well as initial copper metabolism had no impact on the risk of early neurological deterioration (Table 1).

**Fig. 1:**
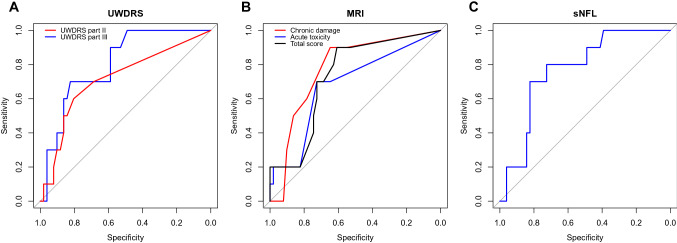
Receiver operating characteristic curves (ROC) for predictors of early neurological deterioration: UWDRS part II and III (**A**), MRI acute toxicity, chronic damage and total brain score (**B**) and sNfL (**C**). MRI, magnetic resonance imaging; sNfL, serum neuro-filament light chain; UWDRS, Unified Wilson’s Disease Rating Scale

UWDRS part III was characterised by the greatest AUC (0.803), with slightly lower AUC values for chronic damage score (0.774) and sNfL (0.770) (Fig. 1; Table 2). UWDRS part III was also characterised by the greatest specificity and PPV, whereas chronic damage score and the total MRI score had the greatest sensitivity (Fig. 1; Table 2). All predictors were characterised by very high NPVs (> 0.9), but PPVs were low (all < 0.5, Table 2).

**Table 2:** Detailed results of receiver operating characteristic curve analyses for predictors of early neurological deterioration

	AUC (95% CI)	Optimal cut-off	Sensitivity (95% CI)	Specificity (95% CI)	PPV (95% CI)	NPV (95% CI)
UWDRS part III	0.803 (0.669–0.916)	17.50	0.700 (0.400–1.000)	0.823 (0.706–0.922)	0.434 (0.266–0.643)	0.935 (0.872–1.000)
UWDRS part II	0.715 (0.530–0.879)	1.50	0.600 (0.300–0.900)	0.804 (0.686–0.902)	0.338 (0.200–0.583)	0.913 (0.848–0.976)
MRI: acute toxicity	0.682 (0.514–0.849)	1.50	0.700 (0.400–1.000)	0.725 (0.588–0.843)	0.333 (0.200–0.500)	0.927 (0.857–1.000)
MRI: chronic damage	0.774 (0.623–0.901)	1.50	0.900 (0.700–1.000)	0.647 (0.509–0.764)	0.333 (0.250–0.435)	0.971 (0.912–1.000)
MRI: total score	0.731 (0.563–0.869)	1.50	0.900(0.700–1.000)	0.608 (0.471–0.745)	0.310 (0.233–0.409)	0.970 (0.903–1.000)
sNfL	0.770 (0.622–0.896)	18.15	0.800 (0.500–1.000)	0.725 (0.588–0.843)	0.363 (0.250–0.5000	0.950 (0.881–1.000)

*AUC* area under the curve, *CI* confidence interval, *MRI* magnetic resonance imaging, *NPV* negative predictive value, *PPV* positive predictive value, *SD* standard deviation, *sNfL* serum neuro-filament light chain, *UWDRS* Unified Wilson’s Disease Rating Scale

Apart from type of treatment, all predictors analysed in the whole group of WD patients by ROC curve analyses were statistically significant in univariate logistic regression models. The greatest risk of deterioration was when the scores were above cut-offs for chronic damage on MRI (OR = 16.50), UWDRS part III (OR = 10.89), and sNfL (OR = 10.57) (see Table 3 for all results). However, when the analysis was performed only in patients with neurological symptoms, we found statistical significance only for sNfL (OR = 6.40); other predictors were associated with increased risk of deterioration (ORs ≥ 2.00), but the effect sizes were lower than in the analysis amongst all patients and were non-significant (OR ≤ 6.40, see Table 3). In bivariate logistic regression models, when corrected for baseline UWDRS part III scores (OR = 7.14), only sNfL remained a significant predictor of worsening (OR = 6.94); the remaining predictors increased the risk of worsening, albeit non-significantly (Table 4). Moreover, sNfL remained a significant predictor of worsening when corrected for all MRI scores (Table 4).

**Table 3:** Univariate logistic regressions predicting neurological deterioration amongst all patients and those with neurological symptoms only

Predictor	All patients (*n* = 61)		Patients with neurological symptoms (*n* = 36)	
	OR (95% CI)	*P* value*	OR (95% CI)	*P* value*
d-penicillamine vs zinc sulphate	1.98 (0.49–1.87)	0.333	1.27 (0.29–5.66)	0.740
UWDRS Part II (above vs below cut-off)	6.15 (1.46–26.00)	0.014	2.40 (0.5–10.6)	0.250
UWDRS Part III (above vs below cut-off)	10.89 (2.35–50.40)	0.002	4.40 (0.9–21.3)	0.065
MRI: acute toxicity (above vs below cut-off)	6.17 (1.40–27.25)	0.016	2.00 (0.4–9.4)	0.383
MRI: chronic damage (above vs. below cut-off)	16.50 (1.93–140.85)	0.010	5.62 (0.6–51.3)	0.616
MRI: total score (above vs. below cut-off)	13.95 (1.64–118.70)	0.016	4.00 (0.4–37.1)	0.431
sNfL (above vs. below cut-off)	10.57 (2.00–55.99)	0.006	6.40 (1.12–36.43)	0.036

Cut-off values for individual predictors were taken from receiver operating characteristic curve analyses

*CI* confidence interval, *MRI* magnetic resonance imaging, *OR* odds ratio, *sNfL* serum neuro-filament light chain, *UWDRS* Unified Wilson’s Disease Rating Scale

^*^Wald test

**Table 4:** Bivariate logistic regression models predicting neurological deterioration amongst all patients

	Predictor (above vs below cut-off)	OR (95% CI)	*P* value^*^
Model 1	sNfL	6.94 (1.19–40.47)	0.031
	UWDRS part III	7.14 (1.40–36.49)	0.018
Model 2	MRI: acute toxicity	1.63 (0.20–13.30)	0.649
	UWDRS part III	7.76 (0.97–62.14)	0.054
Model 3	MRI: chronic damage	7.58 (0.69–83.10)	0.097
	UWDRS part III	4.01 (0.70–22.92)	0.118
Model 4	MRI: total score	5.64 (0.46–68.46)	0.175
	UWDRS part III	4.28 (0.71–25.92)	0.114
Model 5	MRI: acute toxicity	3.88 (0.79–19.09)	0.095
	sNfL	7.64 (1.37–42.82)	0.021
Model 6	MRI: chronic damage	10.32 (1.13–94.24)	0.039
	sNfL	6.31 (1.09–36.63)	0.040
Model 7	MRI: total score	8.22 (0.89–75.57)	0.063
	sNfL	6.30 (1.10–36.16)	0.039

Cut-off values for individual predictors were taken from receiver operating characteristic curve analyses

*CI* confidence interval, *MRI* magnetic resonance imaging, *OR* odds ratio, *sNfL* serum neuro-filament light chain, *UWDRS* Unified Wilson’s Disease Rating Scale

^*^Wald test

## Discussion

Based on the validated UWDRS and defined criteria for neurological deterioration, we found that early neurological deterioration after treatment initiation occurred in 16.3% of patients with WD in our clinic. Interestingly, and as previously described, neurological deterioration occurred only in patients with the neurological phenotype (27.7%; 10/36) at the time of diagnosis and just after initiation of decoppering treatment [14]. Comparing our results with published literature, we found more cases of neurological deterioration in the current cohort than in our previous work (11.1%) or in papers published by Weiss et al. (9.1% on d-penicillamine, 8.8% on trientine and 7.3% on zinc therapy) [23], but less than reported in early studies (up to 50% on d-penicillamine [23] and 30.2% as reported by Kalita et al. [24]). Taking results from Poland and Germany together, the mean percentage of early neurological deterioration in adequately treated WD patients is estimated at 10.5% (range 7.3–16) [14, 23]. As some deteriorated WD patients will not improve over time, these data are also concordant with long-term follow-up studies reporting that 85% of WD patients had good outcome [11].

However, analysing only WD patients with neurological symptoms, the data according to early neurological deteriorations look very similar in this group of patients [14, 23, 24]. Weiss et al*.* analysed mostly hepatic WD patients (only 34.4% patients had neurological symptoms), and the frequency of neurological deterioration was nearly twice as low as in our sample. Kalita et al. [24] analysed mainly neurological WD patients (59/63) and early neurological deterioration occurred in 32.2% of them (19/59). In another study, 22.8% (16/70) of patients with neurological WD had an early deterioration [14]. The frequency of an early deterioration in neurological disease in our study (27.7%) is similar to previously reported figures. Because the risk of deterioration is substantially higher in neurological than non-neurological WD, future studies should analyse the predictors of deterioration separately for neurologic and neurologic phenotypes.

We found that markers of initial brain injury in WD, namely UWDRS score, brain MRI chronic damage score and total score, and sNfL concentrations were predictors of early neurological deterioration amongst patients with all phenotypes. Brain MRI chronic damage score was a stronger predictor of neurological deterioration than the acute toxicity score. At baseline, the mean acute toxicity score was non-significantly greater in patients that worsened after 6 months. Because the deterioration was observed only in patients with neurological symptoms at diagnosis, and only initial sNfL concentrations predicted the neurological deterioration in this subgroup, higher UWDRS and brain MRI scores reflected a greater severity of neurological disease [14, 23, 24, 31].

Together with observations of a lack of effect of anti-copper drugs in some patients, it appears that early neurological deterioration may be occurring as part of the natural course of the disease in certain cases. Previous neurological disease and irreversible chronic damage (as seen by MRI) may make improvements with treatment impossible in these patients. We suggest that a new definition of treatment-induced early neurological deterioration is needed, based on (potentially reversible) clinical as well as neuroradiological aspects, to differentiate from those who deteriorate due to natural progression.

In our study, we also propose cut-off points, based on ROC curves, to establish more practically which patients will deteriorate. These data expand on previous studies performed by Litwin et al., which suggested the significance of severity of neurological disease scored in UWDRS as a predictor of early neurological worsening in WD [14]. Further, our demonstration that sNfL concentration also predicts early neurological deterioration is consistent with other studies that demonstrated that sNfL reflects neuro-axonal injury [25–28] and neural tissue damage in many disorders, as well as even predicting death in SARS-CoV-2 patients [29]. In bivariate logistic regressions for values above the cut-offs, we found that sNfL was a significant risk factor of neurological deterioration independent of UWDRS and MRI. Moreover, sNfL concentration was the only significant predictor of deterioration amongst patients with neurological symptoms only. These findings suggest that sNfL could indicate the severity of ongoing brain injury, giving additional information to clinical and neuroimaging scores, which reflect brain damage that has taken place over longer periods. In line with this view, amongst patients with neurological WD, Shribman et al. found that sNfL concentrations were significantly increased in those with ‘active’ disease (neurological presentation or recent deterioration due to non-adherence) [19].

In our study, we found only statistically significant trend towards longer disease latency (time from symptoms onset to treatment introduction in WD) as a predictor of neurological deterioration. Previous study by Litwin et al. [14] did not find such effect, and other studies evaluating predictors of early neurological deterioration did not include this parameter in analysis [24, 31, 32]. Lack of positive results in this topic may be due to possible problems with definition of age at symptoms onset, which mainly depends on treating physicians’ decision and patients’ data availability [33, 34]. Additionally, neurological symptoms in WD occur usually a few years after hepatic symptoms, so longer disease latency in neurological WD patients may be just a consequence of neurological form of disease. However, we did not find a statistically significant difference in disease latency between hepatic and neurological WD [21].

Further, we did not find any effect of different anti-copper treatments (d-penicillamine or zinc salts) or initial copper metabolism parameters on the risk of early neurological deterioration. Previously, we have reported that historically used high doses of d-penicillamine could lead to neurological deterioration [6]; however, respecting the rule ‘start low and go slow’ now appears to be permitting the safe use of d-penicillamine in WD management.

In the literature, there are various methods of definition of early neurological deterioration in WD. Initially (in retrospective clinical registries), authors reported just subjective information about worsening or not [6, 10, 12]. As discussion about treatment superiority or no-inferiority in WD started and new therapies and drugs were studied, several methods of neurological deterioration in WD were proposed [24, 31, 32]. They included modified Young scale (scoring more than 2 additional points defined deterioration; used in Chinese studies, not adapted for other countries) [31, 32], Burke–Fahn–Marsden scale (increase of more than 10% of points defined worsening; Indian studies and scales dedicated mainly to asses dystonia) [24]. UWDRS was introduced in 2007 [16]. The definition of neurological deterioration used in this paper was first proposed in 2015 [14] and since that time was used in clinical trials in WD [16], what made us to use it in our study. However, based on our results, we suggest that possibly the definition and explanation of this phenomenon should be redefined. Finally, in the available literature, there are few papers which aimed to analyse the risk factors of early neurological deterioration. Those available mainly focussed on WD clinical symptoms as risk factors [14, 24, 31]. Litwin et al. found that concomitant anti-dopaminergic medications, severity of WD neurological disease as well as localization of brain MRI lesions in pons and thalami are risk factors of neurological worsening [14]. Kalita et al. analysed mainly symptomatic neurological WD patients treated with d-penicillamine and found association between deterioration and drooling, evidence of chronic liver disease, splenomegaly, leukopenia and thrombocytopenia [24]. Finally, Hou et al. analysed 47 neurological WD patients treated with d-penicillamine and described the younger age, shorter delay of diagnosis, dystonia and severe mutation genotype as risk factors of deterioration [31]. These observations did not include different types of treatment of the symptomatic WD patients, and were based just on clinical data like age, neurological phenotype, presence of liver cirrhosis and their symptoms, type of mutations, which factors generally affected outcome in several studies with long follow-up [5–7]. Using the new techniques, based on recently published results on biomarkers in WD, our study was the first which documented the significance of quantitative biomarkers of neurological injury as a predictors of neurological deterioration [18, 19, 21, 30]. Our study has several limitations. First, it was performed on a limited number of patients; however, as WD is a rare disease, it is difficult to include large number of patients in a single-centre study where methodologies are all kept the same. Assessments of brain MRI semiquantitative scale and UWDRS were performed by the same, well-trained neuroradiologist and neurologists, respectively. However, in previous papers validating both scales, they achieved very good interrater reliability [16, 18]. Further, despite the fact that we included consecutive newly diagnosed WD patients, we cannot exclude the selection bias according to few confounders like type of WD treatment or phenotypic presentation. In our centre, there is no specific protocol for what drug should be introduced as a first-line treatment in WD patients. Each time decision on which drug to start (d-penicillamine or zinc salts) is made together by treating neurologist and patient, after discussing with patients current scientific data, possible side effects, concurrent drugs regimens, pregnancy planning and costs of both treatments. According to phenotypic presentation, our department is the main reference centre for adult WD patients in Poland; however, we are neurological department. Hence, it is possible that patients with severe hepatic symptoms, eligible for liver transplantation are diagnosed and treated in hepatological centres. Moreover, as the aim of our study was to establish the predictors of early neurological deterioration, we analysed patients during first 6 months of treatment initiation (the time when the early deterioration could occur). We did not analyse subsequent resolution of these phenomenon because this would need longer follow-up—up to 1–2 years, which was not the aim of this study. Additionally, during the long course of the disease, several clinical and laboratory factors (e.g. like compliance, additional disorders) may impact the neurological deterioration and should be included in analysis. From a statistical point of view, our groups of WD patients may be not well balanced. However, WD is rare disease. Moreover, statistical methods like multiplying observations for 10 patients with worsening would be associated with an even stronger bias. What should be mentioned, previous clinical trials analysed neurological deterioration ratio in WD on even smaller samples of patients than ours [16]. Finally, we did not perform prospective assessment of sNfL, which would have been interesting to assess against patient outcome. However, our results did allow us to propose measurable quantitative predictors of early neurological deterioration as well as to establish their cut-off points.

## Conclusion

Results from our study indicate that, in neurological WD, initially high sNfL levels are the main risk factors for neurological deterioration. Additionally, high scores on the UWDRS and brain MRI chronic damage scale in patients who deteriorated reflected a higher severity of neurological disease. However, as these clinical and neuroradiological scales allow us to objectively assess the neurological status of WD patients, we recommend that all WD patients be assessed using these measures before introduction of anti-copper treatment to identify if patients are at high risk of deterioration. Patients at higher risk should be treated particularly carefully: clinicians may consider slower introduction or uptitration of chelators, with more frequent checks of copper metabolism, and more regular neurological and safety assessments. With particular focus on the effect of brain MRI chronic damage score, we suggest performing studies to establish a clear definition of treatment-induced early neurological deterioration in WD based on clinical as well as neuroradiological results. Future research should concentrate on prospective studies with sNfL, brain MRI scale and UWDRS measurement during WD treatment [30] to confirm our findings and hypotheses.
